# MiRNA-30a inhibits AECs-II apoptosis by blocking mitochondrial fission dependent on Drp-1

**DOI:** 10.1111/jcmm.12420

**Published:** 2014-10-06

**Authors:** Cuiping Mao, Jinjin Zhang, Shengcui Lin, Lili Jing, Jing Xiang, Meirong Wang, Bingsi Wang, Pan Xu, Weili Liu, Xiaodong Song, Changjun Lv

**Affiliations:** a Molecular Medicine Research Center, Binzhou Medical UniversityYantai, China; b Department of Respiratory Medicine, Affiliated Hospital to Binzhou Medical UniversityYantai, China; c Department of Pathology, Affiliated Hospital to Binzhou Medical UniversityYantai, China; d Department of Sanitation Management, Binzhou Medical UniversityYantai, China; e Clinical Laboratory, Affiliated Hospital to Binzhou Medical UniversityYantai, China; f Department of Respiratory Medicine, Affiliated Hospital to Binzhou Medical UniversityBinzhou, China

**Keywords:** AECs-II, apoptosis, Drp-1, lung fibrosis, miRNA-30a, mitochondrial fission

## Abstract

Apoptosis of type II alveolar epithelial cells (AECs-II) is a key determinant of initiation and progression of lung fibrosis. However, the mechanism of miR-30a participation in the regulation of AECs-II apoptosis is ambiguous. In this study, we investigated whether miR-30a could block AECs-II apoptosis by repressing mitochondrial fission dependent on dynamin-related protein-1 (Drp-1). The levels of miR-30a *in vivo* and *in vitro* were determined through quantitative real-time PCR (qRT-PCR). The inhibition of miR-30a in AECs-II apoptosis, mitochondrial fission and its dependence on Drp-1, and Drp-1 expression and translocation were detected using miR-30a mimic, inhibitor-transfection method (gain- and loss-of-function), or Drp-1 siRNA technology. Results showed that miR-30a decreased in lung fibrosis. Gain- and loss-of-function studies revealed that the up-regulation of miR-30a could decrease AECs-II apoptosis, inhibit mitochondrial fission, and reduce Drp-1 expression and translocation. MiR-30a mimic/inhibitor and Drp-1 siRNA co-transfection showed that miR-30a could inhibit the mitochondrial fission dependent on Drp-1. This study demonstrated that miR-30a inhibited AECs-II apoptosis by repressing the mitochondrial fission dependent on Drp-1, and could function as a novel therapeutic target for lung fibrosis.

## Introduction

Some researchers support that a series of repetitive micro-injuries to the alveolar epithelium contributes to a pathogenetic cascade that leads to histological changes in lung fibrosis [1,2]. Type II alveolar epithelial cells (AECs-II), known as a kind of alveolar epithelium cell, are multifunctional cells involved in surfactant synthesis and secretion, fluid transport and recovery from lung injury. The integrity of AECs-II is indispensable for the maintenance of pulmonary function. Currently available data suggest that the apoptosis of AECs-II caused by injury, such as oxidative stress, is a key determinant of initiation and progression of lung fibrosis [3,4]. MiRNAs have emerged in the recent decade as key regulators of organ phenotypes and potential targets for therapeutic interventions in multiple diseases and gained more and more attention in lung fibrosis. Approximately 10% of the miRNAs are significantly changed in idiopathic pulmonary fibrosis. Many of them later were verified and were shown to have a potential role in the disease [5]. Epigenetic silencing of miR-17–92 occurred in lung tissue and fibroblast cell lines from patients with idiopathic pulmonary fibrosis as a result of enhanced DNA methylation [6]. MiR-199a-5p is up-regulated during fibrogenic response to tissue injury and mediates TGFβ-induced lung fibroblast activation by targeting caveolin-1 [7]. MiR-375 regulates rat alveolar epithelial cell trans-differentiation by inhibiting Wnt/b-catenin pathway [8]. However, the mechanism underlying the protective effects of miR-30a on AECs-II in lung fibrosis remains unclear.

Mitochondrial fusion and fission are critically involved in maintaining a functional mitochondrion [9]. Most previous studies have revealed that abnormal mitochondrial fusion and fission participate in the regulation of apoptosis. Mitochondrial fusion can inhibit apoptosis, whereas mitochondrial fission can promote the latter [10]. However, whether mitochondrial fission participates in AECs-II apoptosis remains unknown. Mitochondrial fission is stimulated by dynamin-related protein-1 (Drp-1). Drp-1 is recruited from the cytoplasm to the mitochondria when excessive mitochondrial fission occurs [11,12]; but, whether Drp-1 translocation is involved in the process of AECs-II mitochondrial fission remains ambiguous.

To date, no study has addressed the mechanism of miR-30a in affecting apoptosis through mitochondrial fission in lung fibrosis. Our previous study has reported that AECs-II apoptosis was characterized by changes in mitochondria morphology [13]. In the present study, we aimed to determine whether miR-30a could inhibit mitochondrial fission–induced AECs-II apoptosis dependent on Drp-1.

## Materials and methods

### Human tissue samples

The patients of lung fibrosis were from Binzhou Medical University Hospital. Lung tissue samples were obtained from surgical remnants of biopsies. Control non-pulmonary fibrosis was obtained through the Binzhou Medical University Hospital from samples resected from patients with lung cancer. The protocol was approved by the Institutional Review Board of the University of Binzhou Medical University.

### Ethics statement

Sprague–Dawley (SD) rats (8–12 weeks old) were provided by the Yantai Green Leaf Experimental Animal Center (Yantai, China). Sixty SD rats were randomly divided into 6 groups (10 rats each) including the sham group and bleomycin (BLM)-induced groups (3, 7, 14, 21 and 28 days). The rats used in this study were treated in accordance with the Chinese Institutes of Health Guide for the Care and Use of Laboratory Animals.

### Animal model

Pharmaceutical grade BLM was purchased from Nippon Kayaku (Tokyo, Japan). Rats in BLM-induced groups were administered 5 mg/kg BLM dissolved in saline *via* a single intratracheal instillation under anaesthesia as our previously described [14]. The sham group rats were administered an equal volume of saline. Lung tissues were harvested respectively on 3, 7, 14, 21, 28 days following treatment with BLM.

### Cell model

A549 cell line, a human lung epithelium-derived cell line, is commonly used in studies that focus on the function of human AECs-II because this cell line retains the features and metabolic characteristics of AECs-II [15]. A549 cell lines were purchased from the Cell Bank of the Chinese Academy of Sciences (Shanghai, China). Cells were maintained in 1640 medium (Hyclone Co.) containing 10% newborn calf serum, 100 U/ml penicillin and 100 μg/ml streptomycin at 37°C under a humidified atmosphere of 5% CO_2_ and 95% air. Cells were subcultured at an initial density of 1 × 10^5^/ml every 3–4 days. A549 were treated with H_2_O_2_ and harvested respectively on 3, 6, 12, 24 hr following treatment with H_2_O_2_.

### Histopathological examination

After 2 weeks of fixing in paraformaldehyde, lung tissues were embedded in paraffin.

Transverse sections of 4-μm thick slices were stained with haematoxylin and eosin (Sigma-Aldrich, St. Louis, MO, USA) and Masson's trichrome (Maxin Company, Fuzhou, China) stain. The severity of fibrosis was blindly determined by a semiquantitative assay based on previous studies [16,17].

### Sulforhodamine B assay

Cells were digested with 0.25% trypsin at the logarithmic growth phase and diluted to 1 × 10^5^ cells/ml. A quantity of 100 μl media was seeded into 96-well culture plates. After overnight incubation, the cells were incubated with media containing different concentrations of H_2_O_2_ for another 12 hrs. Next, 50 μl of trichloroacetic acid was added to each well for an additional hour at 4°C. Each plate was washed five times with double distilled water and dried under room temperature. The trichloroacetic acid-fixed cells were stained with 100 μl of 0.4% sulforhodamine B (SRB) for 10 min., after the plate was washed five times with 1% acetate and air-dried overnight. The resulting crystals formed were dissolved in 150 μl of 10 mM Tris aminomethane hydrochloride. Absorbance was measured with an ELISA reader (SpectraMax M2, USA) using the wavelength of 540 nm. The inhibitory activity of H_2_O_2_ was expressed in terms of the median inhibitory concentration (IC_50_) value as our previously described [18].

### Quantitative real-time PCR (qRT-PCR)

Total RNA was isolated using TRIzol reagent (Invitrogen, Carlsbad, CA, USA). RNA quantity and quality were measured using the NanoDrop 2000 spectrophotometer (Thermo Scientific, Waltham, MA, USA) and RNA integrity was assessed by standard denaturing agarose gel electrophoresis. Complementary DNA synthesis was performed with the M-MLV reverse transcriptase kit (Invitrogen) following the manufacturer's instructions. qRT-PCR was performed with a SYBR green-based PCR master mix kit (Takara, Shiga, Japan) on a Rotor Gene 3000 real-time PCR system from Corbett Research (Sydney, Australia). The PCR conditions were as follows: initial denaturation at 95°C for 30 sec. followed by 40 cycles of 95°C for 5 sec., and 60°C for 20 sec. Fluorescence signal was monitored at 585 nm during each extension. Glyceraldehyde 3-phosphate dehydrogenase (GAPDH) and U6 served as an internal control.

### Terminal deoxynucleotidyl transferase-mediated dUTP nick-end-labelling (TUNEL) assay

Lung tissues were fixed in 4% paraformaldehyde instilled through the trachea and embedded in paraffin. Transverse sections of 4 μm thickness were prepared. Apoptosis was assessed by TUNEL assay using a kit from KeyGEN BioTECH (Nanjing, China) following the kit's instructions and detected under a laser scanning confocal microscope from Leica Company (Germany).

### Immunofluorescence

Lung tissue sections were fixed in 4% paraformaldehyde, rinsed with PBS solution, incubated with 0.5% Triton X-100 for 15 min. at room temperature, and blocked with 10% serum. Primary antibodies included anti-SP-C; anti-Drp-1 and anti-Bax (Santa Cruz-Watsonville, CA, USA) were added at 4°C overnight. Tissue sections were subsequently rinsed with PBS and incubated with fluorescein labelled IgG antibody SP-C (Texas Red- labelled or FITC-labelled), Drp-1(Cy3-labelled) and Bax (FITC-labelled or Cy3-labelled). Hoechst 33258 (Sigma-Aldrich) was used for nuclear staining. After washing with PBS, tissue sections were mounted in glycerine and detected under a laser scanning confocal microscope from Leica Company.

### Apoptosis analysis

1 × 10^6^ cells/ml was seeded in six-well plates. Suspended and adherent cells were collected and washed with cold PBS. The fixation fluid was washed with PBS as well. Five hundred microlitres of binding buffer was added to resuspend the cells. Five microlitres Annexin V-FIFC and 5 μl propidium iodide staining solution were added for 20 min. away from light. The apoptotic rate was measured by flow cytometry (Beckman, USA).

### Transfections of miR-30a mimic, inhibitor and siRNA

Mimic was used for promote miR-30a expression; inhibitor has the opposite function. MiR-30a mimic, inhibitor and Drp-1 siRNA were synthesized by RiboBio Co.Ltd (Guangzhou, China) for transfection. An amount of 1 × 10^5^ cells were seeded in 24-well plates and cultivated with 1640 medium containing 10% newborn calf serum for 24 hrs. 1.25 μl of 20 μM miR-30a mimic, 1.25 μl of 20 μM Drp-1 siRNA or 2.5 μl of 20 μM miR-30a inhibitor was dilute with 50 μl 1× ribo*FECT*™ CP buffer and incubated for 5 min. at room temperature. Five microlitres ribo*FECT*™ CP reagents were added and incubated for 15 min. at room temperature. The mixed liquor was added to 443.75 μl 1640 medium without 10% newborn calf serum. Cells were incubated with the mixed liquor for 48 hrs. Drp-1 siRNA sense sequence is 5′-GAGGAGAGCUCUUCGAUUU dTdT-3′, Drp-1 siRNA antisense sequence is 3′-dTdT CUCCUCUCGAGAAGCUAAA-5′.

### Observation of ultrastructure under transmission electron microscopy

Lung tissues were fixed in fresh 3% glutaraldehyde for at least 4 hrs at 4°C, post-fixed in 1% osmium tetroxide for 1.5 hrs, dehydrated in a gradient series of ethanol, infiltrated with Epon 812, embedded and cultured at 37, 45 and 60°C for 24 hrs. Ultrathin sections were ultracut using an ultracut E ultramicrotome and stained with uranyl acetate and lead citrate prior to observation under a JEM-1400 transmission electron microscopy (TEM; Jeol Ltd).

### Mitochondrial staining

Cells were plated onto culture dishes or the cover slips coated with 0.01% poly-l-lysine. After treatment, the cells were washed with PBS for three times and fixed in 4% paraformaldehyde for 1 hr at room temperature, then incubated with 0.5% Triton X-100 for 15 min. and stained for 40 min. with 100 nM MitoTracker Red CMXRosb (Molecular Probes) at 37°C. Samples were visualized using laser scanning confocal microscope (Leica).

### Mitochondria isolation

Mitochondria were isolated using a mitochondria isolation kit from Sangon Biotech (Shanghai, China) according to the manufacturer's instructions. Cytoplasmic extraction buffer was used in cytoplasmic protein extraction and mitochondrial protein was isolated by mitochondrial extraction buffer. Protein was denatured and analysed by western blotting.

### Western blot analysis

Protein concentration was quantified using a bicinchoninic acid protein assay kit and boiled with the sample buffer in a water bath for 5 min. Protein samples were separated with 15% SDS-PAGE gels for 2 hrs and transferred onto a polyvinylidene difluoride membrane, which was subsequently blocked in 5% non-fat milk for 2 hrs. Blots were probed using the primary antibodies. The anti-Drp1 antibody was from Santa Cruz Biotechnology. After three times washing with tris buffered saline tween, the horseradish peroxidase-conjugated secondary antibodies were added. Antigen-antibody complexes were visualized by enhanced chemiluminescence.

### Statistical analysis

Data were expressed as the mean ± SEM from the indicated number of independent experiments. Statistical analysis was performed with spss 11.0 software by one-way anova and Student's *t*-test. Correlation analysis was explored by Spearman's correlation coefficient. A *P* < 0.05 was considered to indicate a statistically significant difference.

## Results

### MiR-30a expression decreased *in vivo* and *in vitro* models of lung fibrosis

**Fig. 1:**
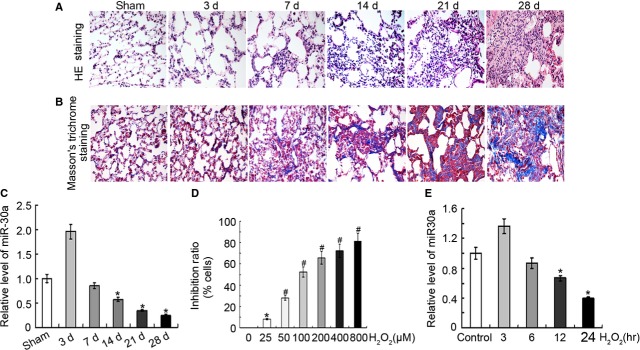
MiR-30a decreased *in vivo* and *in vitro* models of lung fibrosis. (**A**) Haematoxylin and eosin staining showed that the severity of the pathology of lung fibrosis increased in BLM-induced group, magnification ×400. (**B**) Masson's trichrome staining indicated that collagen increased in BLM-induced group, magnification ×400. (**C**) The qRT-PCR analysis demonstrated that miR-30a decreased in BLM-induced lung fibrosis. (**D**) Inhibitory effect of H_2_O_2_ was detected using an SRB assay. (**E**) The qRT-PCR analysis showed that miR-30a decreased in H_2_O_2_-induced group. A549 was treated with 120 μM H_2_O_2_, and harvested at 3, 6, 12, and 24 hrs. Each bar represents the mean ± SD, *n* = 6, *P < 0.05; ^#^*P* < 0.01.

Animal models were detected through haematoxylin and eosin staining and Masson's trichrome staining (Fig. 1A and B). Result showed that alveolar structure was complete and continuous without significant inflammation and that the alveolar septum was thinner and has few collagen fibres in sham group. On the contrary, the alveolar structure was damaged with alveolar walls thickening and oedema occurred and a significant number of collagen fibres were deposited in the BLM-induced groups. These results indicated that a lung fibrosis model induced by BLM was successfully established.

The qRT-PCR analysis demonstrated that miR-30a levels significantly decreased in the BLM-treated rat model (Fig. 1C). Interestingly, the expression of miR-30a in the 3 days group increased compared with the sham group. MiR-30a started to down-regulate in the rat treated with BLM for 7 days, and the miR-30a was fallen to the lowest level at 28 days. With the increase in the severity of lung fibrosis, the expression of miR-30 decreased.

Then, we also analysed the level of miR-30a *in vitro*. A549 was exposed to graded doses of H_2_O_2_ (0–800 μM) for 12 hrs. A dose-dependent inhibitory effect was observed. The IC_50_ value in A549 was 92.87 μM (Fig. 1D). The proliferation inhibition rate of A549 treated with 120 μM H_2_O_2_ was 59.10%. About 120 μM H_2_O_2_ was used to evaluate miR-30a *in vitro* and promote the inhibitory effect of H_2_O_2_. The qRT-PCR analysis demonstrated that miR-30a levels significantly reduced in H_2_O_2_-treated A549. Interestingly, the expression of miR-30a increased in the H_2_O_2_-treated group for 3 hrs. The level of miR-30a started to decline at 6 hrs, and the level of miR-30a reached the lowest point at 24 hrs (Fig. 1E). The trend of miR-30a expression *in vitro* was similar to that *in vivo*.

### MiR-30a inhibited AECs-II apoptosis

**Fig. 2:**
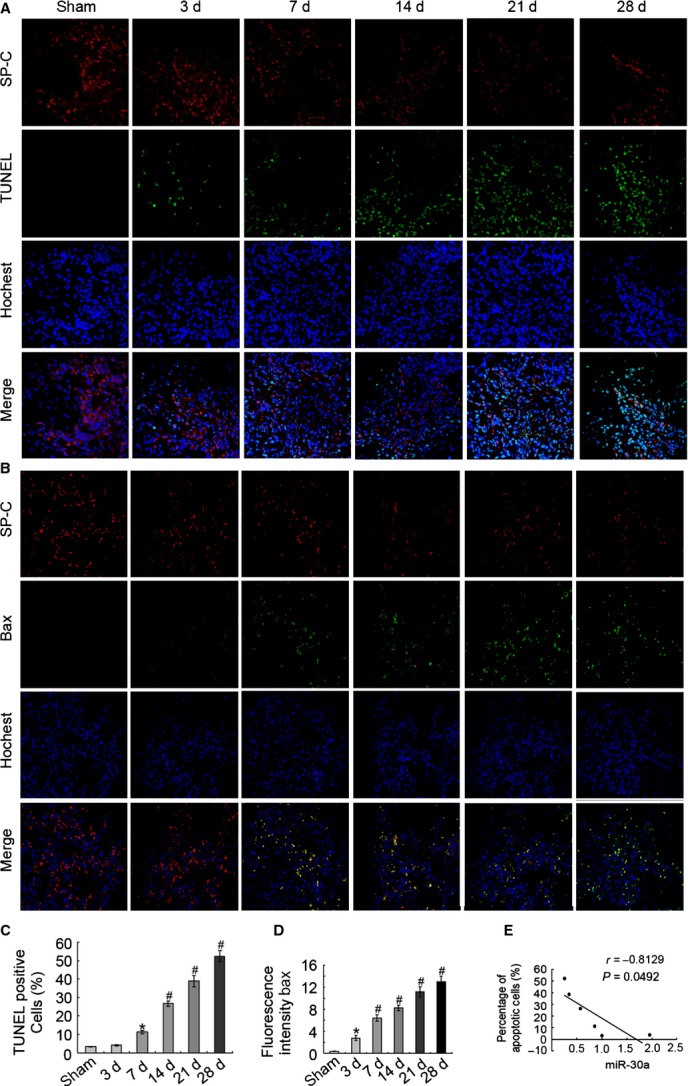
AECs-II apoptosis increased *in vivo* models of lung fibrosis. (**A**) Apoptotic cell nucleus was stained using TUNEL labelled with FITC (green). The SP-C and nucleus were stained with SP-C antibody (red) and Hochest 33258 (blue), respectively. (**B**) SP-C and Bax were stained with SP-C antibody (red) and Bax antibody (green), respectively, whereas the nucleus was stained with Hoechst 33258 (blue). (**C**) TUNEL-positive cells increased *in vivo*, and the TUNEL^+^ SPC^+^ cells and SPC^+^ cells in each whole image were counted; the bar graph represents the rate of TUNEL^+^SPC^+^ cells in SPC^+^ cells from three independent experiments. (**D**) The expression of Bax increased *in vivo*. The mean fluorescence intensity of Bax in each whole image was automatically quantified using Image-Pro Plus software and expressed in fluorescence units (FU); the bar graph represents the average value from three independent experiments. (**E**) MiR-30a was inversely correlated with apoptotic cells, *r* = −0.8129. Each bar represents the mean ± SD *n* = 6, *P < 0.05; ^#^*P* < 0.01.

**Fig. 3:**
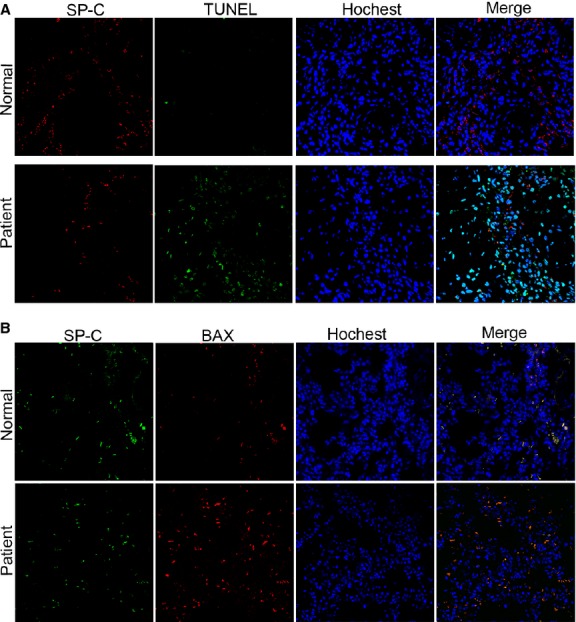
AECs-II apoptosis increased in the lung fibrosis of the patient. (**A**) Apoptosis was detected through TUNEL. Apoptotic cell nucleus and SP-C were stained with TUNEL labelled with FITC (green) and SP-C antibody (red). (**B**) SP-C and Bax expression was determined using a laser scanning confocal microscope. SP-C and Bax were stained, respectively, with SP-C antibody (green) and Bax antibody (red), nuclei was stained by hochest 33258 (blue).

AECs-II apoptosis were evaluated through TUNEL. TUNEL-positive cells (condensed and fragmented nuclear DNAs) were increased about 11.5-fold in the BLM-induced 28 days lung fibrosis compared with the sham group. Furthermore, SP-C (the marker of AECs-II) was stained to confirm that AECs-II underwent apoptosis. The merged results showed that these injured DNAs were observed in the cell nucleus of AECs-II. Thus, AECs-II apoptosis existed in the rat model of lung fibrosis (Fig. 2A and C). The expression of Bax also increased in BLM-induced lung fibrosis (Fig. 2B and D). TUNEL-positive cells and Bax expression increased in the sample with lung fibrosis patients (Fig. 3). These data are supported by the obtained results from our previous research on patients, indicating that the present study was clinically significant for lung fibrosis [13]. The level of miR-30a was inversely correlated with the apoptotic cells *in vivo*. With prolonged BLM induction, the expression of miR-30a decreased; conversely, the apoptotic cells increased (Fig. 2E).

**Fig. 4:**
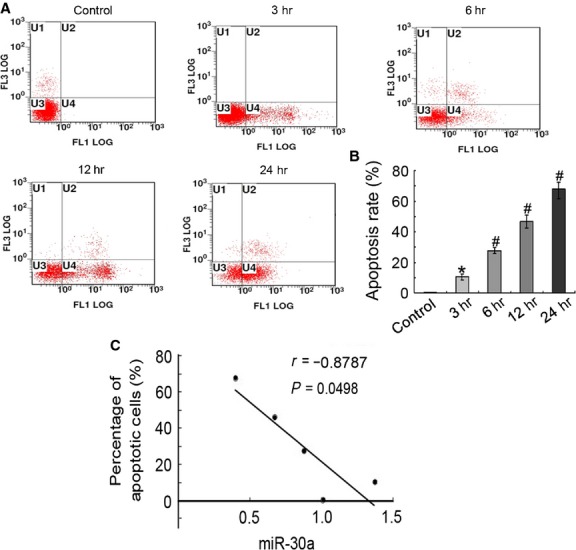
AECs-II apoptosis increased *in vitro* models of lung fibrosis. (**A**) Apoptosis was detected through flow cytometry. U1 represents necrotic cells; U2 denotes middle-late apoptotic cells; U3 indicates normal cells; U4 denotes early apoptotic cells. U2+U4 represents apoptotic cells. A549 was treated with 120 μM H_2_O_2_ and obtained at 3, 6, 12, and 24 hrs. With the extension of H_2_O_2_-induced time, apoptotic cells increased. (**B**) Apoptotic rate statistics of each group. (**C**) MiR-30a was inversely correlated with apoptotic cells. *r* = −0.8787. Each bar represents the mean ± SD, *n* = 6, *P < 0.05; ^#^*P* < 0.01.

For the limitations of miR-30a transfection in animals, a cell model was established to further study the effect of miR-30a on apoptotic AECs-II. The results showed that apoptotic rate increased with the extension of H_2_O_2_-induced time and reached 50% when H_2_O_2_ was treated for 12 hrs. Thus, H_2_O_2_ treatment for 12 hrs was used to further study the effect of miR-30a *in vitro* (Fig. 4A and B). The level of miR-30 was inversely correlated with the apoptosis of A549 treated with H_2_O_2_. With the extension of H_2_O_2_-induced time, the level of miR-30 decreased; conversely, the apoptotic A549 increased (Fig. 4C).

**Fig. 5:**
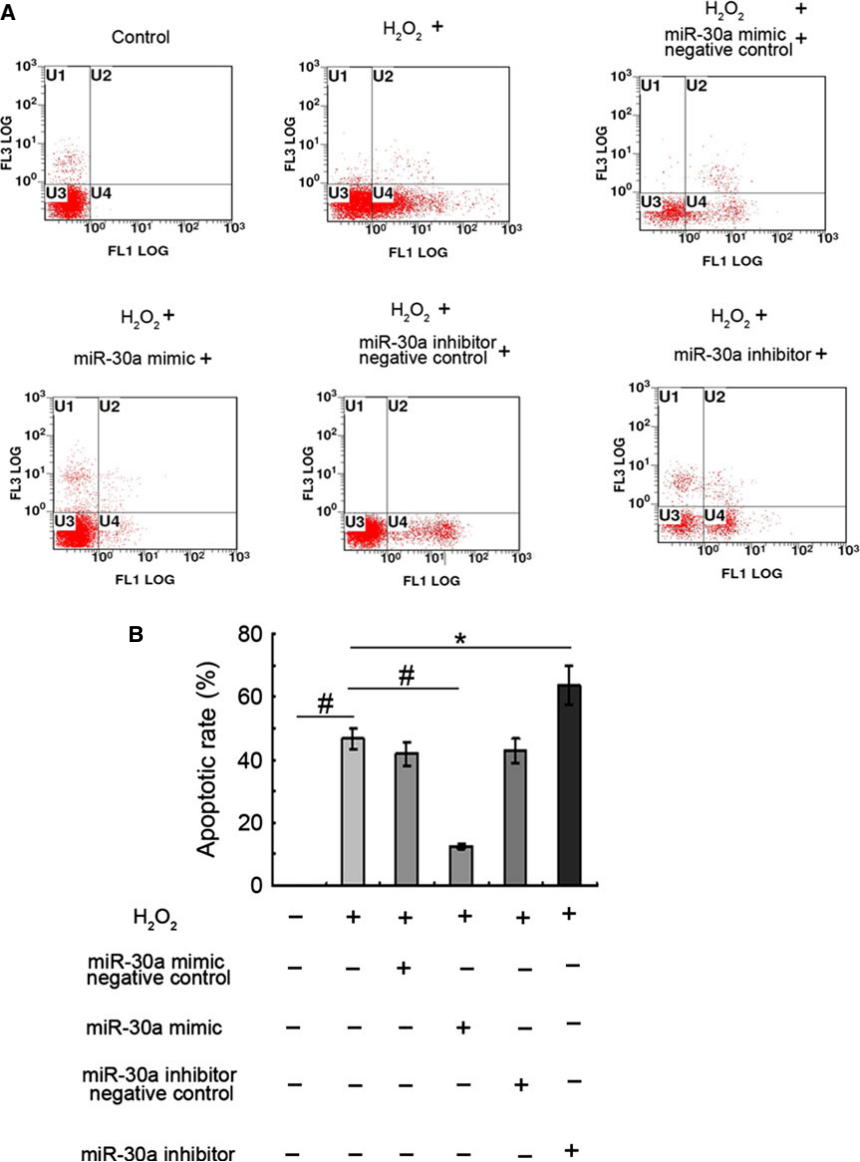
MiR-30a inhibited AECs-II apoptosis. (**A**) A549 was transfected with miR-30a mimic-negative control or mimic at 50 nM for 48 hrs, and then treated with H_2_O_2_ for 12 hrs; A549 was transfected with miR-30a inhibitor-negative control or inhibitor at 100 nM for 48 hrs, and then co-treated with H_2_O_2_ for 12 hrs. (**B**) Apoptotic rate of each group. Each bar represents the mean ± SD, *n* = 6, *P < 0.05; ^#^*P* < 0.01.

A549 was transfected with miR-30a mimic (gain-of-function) and inhibitor (loss-of-function) to explore the effect of miR-30a on AECs-II apoptosis. The flow cytometry result showed that miR-30a mimic made a 2.67-fold decrease in the apoptotic rate. While miR-30a inhibitor could aggravate H_2_O_2_-induced A549 apoptosis (Fig. 5), gain- and loss-of-function studies revealed that down-regulation of miR-30a could promote A549 apoptosis. Thus, these results revealed that miR-30a could inhibit AECs-II apoptosis.

### MiR-30a inhibited mitochondrial fission

**Fig. 6:**
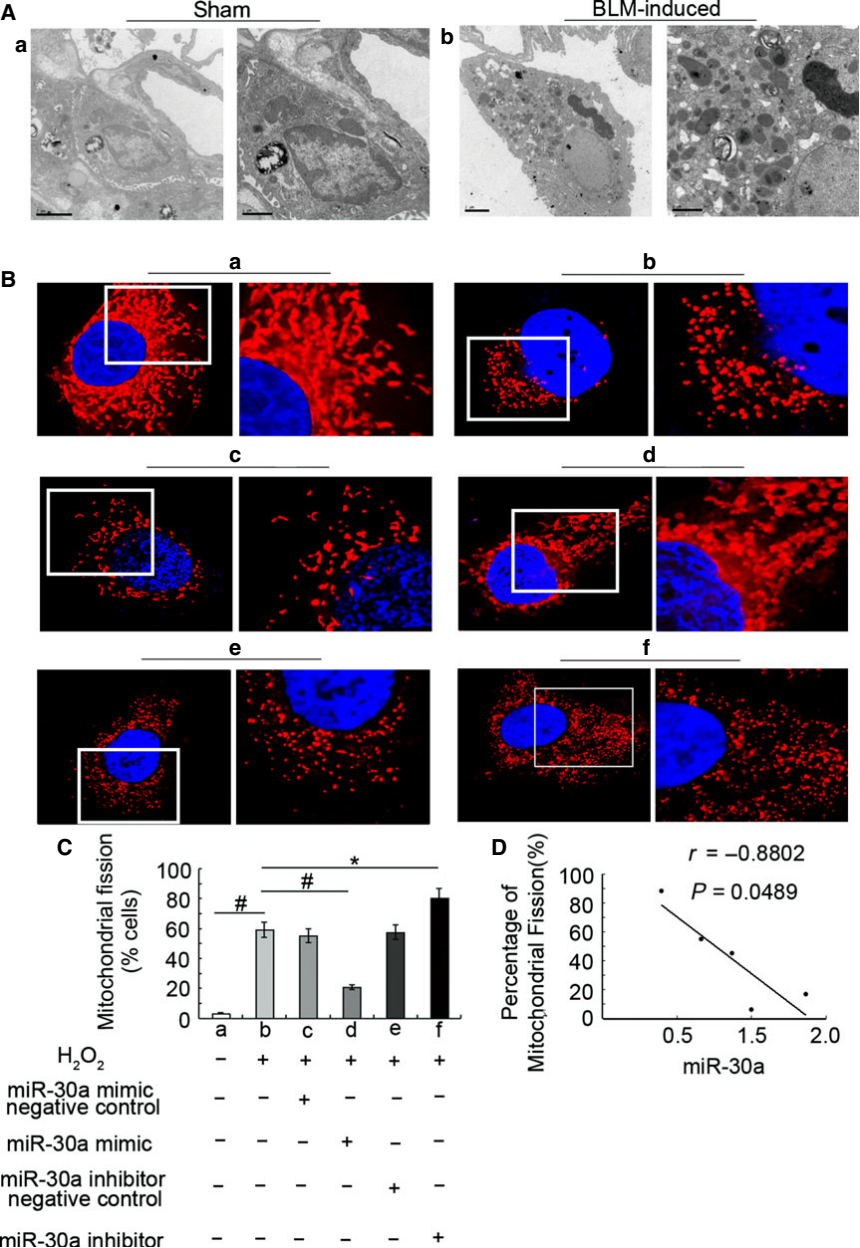
MiR-30a inhibited mitochondrial fission *in vivo* and *in vitro* models of lung fibrosis. (**A**) Mitochondrial fission was detected through TEM. Mitochondria became smaller and increased in BLM-induced group compared with the sham group. (**a**) Sham group, (**b**) BLM-induced group. (**B**) Mitochondrial morphology was observed using a laser scanning confocal microscope. (**a**) Control group, (**b**) 12 hrs H_2_O_2_-induced group, (**c** and **d**) A549 were transfected with miR-30 mimic-negative control or mimic at 50 nM for 48 hrs, respectively, and then co-treated with H_2_O_2_ for 12 hrs. (**e** and **f**) A549 was transfected with miR-30 inhibitor-negative control or inhibitor at 100 nM for 48 hrs and then co-treated with H_2_O_2_ for 12 hrs. (**C**) Percentage of mitochondrial fission cells. (**D**) miR-30a was inversely correlated with mitochondrial fission cells. *r* = −0.8802. Each bar represents the mean ± SD, *n* = 6, *P < 0.05; ^#^*P* < 0.01.

Transmission electron microscopy results showed that the morphology of the mitochondria was long and filamentous in AECs-II of the sham group. Mitochondria became shorter and smaller, and the number of mitochondria increased in AECs-II which displaying apoptotic feature such as mushroom-like protrusions in lung tissue of fibrotic rats (Fig. 6A). These data showed that mitochondrial fission possibly participated in AECs-II apoptosis. Next we used gain- and loss-of-function studies to explore the effect of miR-30a on mitochondrial fission. The mitochondria had a tubular network structure in the control group and gain-of-miR-30a function group, but a fragmented structure in the H_2_O_2_-treated and loss-of-miR-30a function group (Fig. 6B). Mitochondrial fission cells were decreased about 1.85-fold in gain-of-miR-30a function group, compared to H_2_O_2_ treatment group. Loss-of-miR-30a function could increase mitochondrial fission cells (Fig. 6C). Correlation analysis showed that miR-30a was inversely correlated with mitochondrial fission cells. With prolonged H_2_O_2_ induction, the expression of miR-30a decreased; conversely, mitochondrial fission cells increased (Fig. 6D). Thus, this finding showed that miR-30a could inhibit mitochondrial fission.

### MiR-30a inhibited Drp-1 expression

**Fig. 7:**
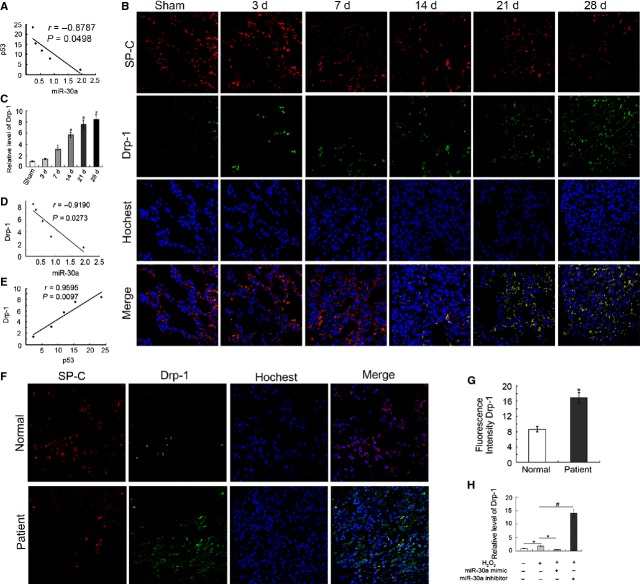
MiR-30a inhibited Drp-1 expression. (**A**) MiR-30a was inversely correlated with p53. *r* = −0.8787. (**B**) Immunofluorescence showed that Drp-1 expression increased *in vivo*. Drp-1 expression was determined using a laser scanning confocal microscope. SP-C (red) and Drp-1 (green) were stained with IgG labelled with Cy3 and IgG labelled with FITC, respectively. Nuclei (blue) were counter-stained with Hoechst 33258. (**C**) The qRT-PCR analysis demonstrated that Drp-1 expression increased *in vivo*. (**D**) MiR-30a was inversely correlated with Drp-1 expression. *r* = −0.9190. (**E**) p53 was positive correlated with Drp-1. *r* = 0.9595. (**F**) SP-C and Drp-1 expression were determined using a laser scanning confocal microscope in the patient of lung fibrosis. SP-C, Drp-1 and nucleus was stained as A. (**G**) Drp-1 expression increased in the lung fibrosis of the patient. (**H**) Drp-1 expression was detected using qRT-PCR *in vitro*. Each bar represents the mean ± SD, *n* = 6, *P < 0.05; ^#^*P* < 0.01.

Li *et al*. reported that p53 is a potential target of miR-30a [10]. Correlation analysis showed that miR-30a was inversely correlated with the level of p53 (Fig. 7A). With extension BLM induction, the expression of miR-30a decreased; conversely, the p53 expression increased. Drp-1 expression was determined using immunofluorescence and qRT-PCR analysis. The immunofluorescence data showed that the expression of Drp-1 increased in the rat model of lung fibrosis (Fig. 7B). The results of qRT-PCR demonstrated that the level of Drp-1 significantly increased and reached the highest point in the 28 days group (Fig. 7C). The level of miR-30a was inversely correlated with the Drp-1 expression *in vivo*. With the extension of BLM induction, the level of miR-30 decreased; conversely, the Drp-1 expression increased (Fig. 7D). The level of p53 was positive correlated with the Drp-1 expression. With prolonged BLM induce, the expression of p53 increased; meanwhile, the level of Drp-1 was also increased (Fig. 7E). Interestingly, Drp-1 expression also increased in patients with lung fibrosis compared with the normal sample (Fig. 7F and G). The specific guiding significance for clinical lung fibrosis was further confirmed. A549 was transfected with miR-30a mimic and inhibitor to investigate the effect of miR-30a on Drp-1 expression. The results of qRT-PCR showed that Drp-1 expression increased in the H_2_O_2_-induced group. The miR-30a mimic decreased Drp-1 expression, whereas miR-30a inhibitor increased the expression (Fig. 7H).

### MiR-30a inhibited Drp-1 translocation

**Fig. 8:**
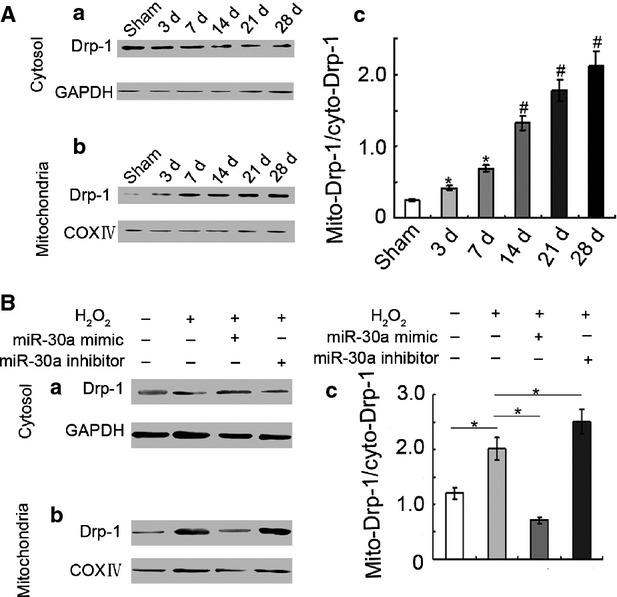
MiR-30a inhibited Drp-1 translocation. (**A**) Drp-1 translocation was detected using western blot *in vivo*. (**a**) Drp-1 and GAPDH expressions in the cytoplasm. (**b**) Drp-1 and COX IV expressions in the mitochondria. (**c**) The rate of Mito-Drp-1 in cyto-Drp-1 increased. (**B**) Drp-1 translocation was determined using western blot *in vitro*. (**a**) Drp-1 and GAPDH expressions in the cytoplasm. (**b**) Drp-1 and COX IV expressions in the mitochondria. (**c**) The rate of Mito-Drp-1 in cyto-Drp-1 decreased in miR-30a mimic-transfection group. The rate of Mito-Drp-1 in cyto-Drp-1 increased in H_2_O_2_-induced group and miR-30a inhibitor-transfection group. Each bar represents the mean ± SD, *n* = 6, *P < 0.05; ^#^*P* < 0.01.

The mitochondria were isolated to demonstrate that Drp-1 translocation existed in lung fibrosis. Western blot showed that mito-Drp-1 increased with the extension of BLM-induced time, compared with cyto-Drp-1. This finding showed that Drp-1 translocated from the cytoplasm to the mitochondria in lung fibrosis (Fig. 8A).

Gain- and loss-of-miR-30a function was used to determine the effect of miR-30a on Drp-1 translocation. Western blot showed that Drp-1 translocated from the cytoplasm to the mitochondria in the H_2_O_2_-induced group, miR-30a mimic repressed the Drp-1 translocation, and miR-30a inhibitor promoted Drp-1 translocation (Fig. 8B).

### MiR-30a inhibited mitochondrial fission and AECs-II apoptosis dependent on Drp-1

**Fig. 9:**
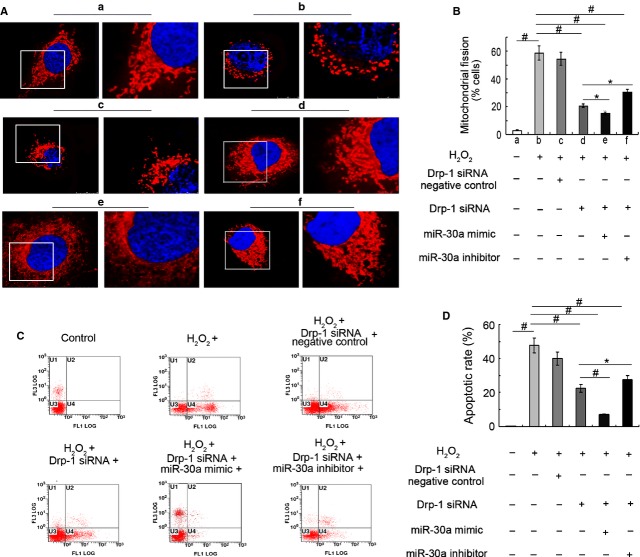
MiR-30a inhibited mitochondrial fission dependent on Drp-1 *in vitro*. (**A**) Mitochondrial morphology was determined with a laser scanning confocal microscope. (**a**) Control group. (**b**) 12 hrs H_2_O_2_-induced group. (**c** and **d**) A549 was transfected with Drp-1 siRNA negative control and Drp-1 siRNA, respectively, at 50 nM for 48 hrs, and then treated with H_2_O_2_ for 12 hrs. (**e**) A549 was cotransfected with Drp-1 siRNA and miR-30a mimic at 50 nM for 48 hrs, and then treated with H_2_O_2_ for 12 hrs. (**f**) A549 was cotransfected with Drp-1 siRNA and miR-30a inhibitor at 100 nM for 48 hrs, and then treated with H_2_O_2_ for 12 hrs. (**B**) The percentage of mitochondrial fission cells. (**C**) Apoptosis was detected using flow cytometry under different conditions. (**D**) Apoptotic rate of each group. Each bar represents the mean ± SD, *n* = 6, *P < 0.05; ^#^*P* < 0.01.

To demonstrate that miR-30a inhibited Drp-1-dependent mitochondrial fission, we used RNAi technology to knock down Drp-1. Drp-1 siRNA caused a 1.83-fold decrease in mitochondrial fission cells. MiR-30a mimic could promote the inhibition effect on mitochondrial fission of Drp-1 siRNA. However, miR-30a inhibitor has the opposite effect. (Fig. 9A and B). The percentage of the mitochondrial fission cells decreased in Drp-1 siRNA and miR-30a inhibitor co-transfection cells compared with miR-30a inhibitor-transfection cells (Fig. 6). Drp-1 siRNA could protect the cell from mitochondrial fission induced by H_2_O_2_. The promoting effect on mitochondrial fission of miR-30a inhibitor decreased. This finding showed that miR-30a inhibited mitochondrial fission.

Drp-1 RNAi technology was used to confirm that miR-30a inhibited Drp-1-dependent AECs-II apoptosis. Apoptotic rate was decreased about 1.12-fold in the Drp-1 siRNA group, compared with H_2_O_2_-treated 12-hr group. Gain-of-miR-30a function could promote the inhibition effect on apoptotic of Drp-1 siRNA. However, loss-of-miR-30a function has the opposite effect (Fig. 9C and D). Apoptotic rate decreased in the Drp-1 siRNA and miR-30a inhibitor co-transfection group compared with the miR-30a inhibitor-transfection group (Fig. 5). Drp-1 siRNA could reverse miR-30a inhibitor-induced apoptosis. These data showed that miR-30a inhibited mitochondrial fission and AECs- II apoptosis dependent on Drp-1.

## Discussion

The miR-30 family is evolutionarily conserved and consists of five members: miR-30a, 30b, 30c, 30d and 30e [19]. The members of the miR-30 family are localized on chromosomes 1, 6 and 8. These members have similar ‘seed sequence’ in their 5′ terminuses. Numerous studies have demonstrated that these members are involved in several cellular processes, such as modulating endothelial cell behaviour during angiogenesis, promotes mitochondria-dependent intrinsic apoptosis and targeting the oncogenic Wnt/β-catenin/BCL9 pathway [20–22]. Pandit *et al*. explored the expression of miRNAs in idiopathic pulmonary fibrosis. Among the significantly decreased miRNAs in idiopathic pulmonary fibrosis were let-7d, miR-26, and miR-30 family [23]. Nevertheless, the antifibrotic mechanism of miR-30a in lung fibrosis remains ambiguous. In this study, we researched the antifibrotic mechanism of miR-30a in AECs-II apoptosis in lung fibrosis. Our data showed that miR-30a might inhibit AECs-II apoptosis by blocking mitochondrial fission dependent on Drp-1.

AECs-II, known as the progenitor cells of the lung epithelium in the alveoli, rapidly proliferates and differentiates into alveolar type I cells after epithelial cell injury. The loss of AECs-II has a significant function in the development and progression of lung fibrosis [24]. Bhandary *et al*. found that p53-mediated inhibition of urokinase-type plasminogen activator and induction of plasminogen activator inhibitor-1 contributes to AECs-II apoptosis that precedes the development of lung fibrosis [25]. Lu *et al*. reported that methylmercury chloride induces AECs-II damage through an oxidative stress-related mitochondrial cell death pathway [26]. Our data showed that AECs-II accumulation occurred during apoptosis in lung fibrosis and up-regulation of miR-30a could inhibit AECs-II apoptosis.

Mitochondria are dynamic organelles that continually undergo fusion and fission [27]. Disordered mitochondrial fission and fusion have been increasingly recognized as contributors to the pathogenesis of diseases, such as cancer, cardiovascular diseases, neurodegenerative diseases and diabetes [28–34]. Over the past 20 years, many connections have been discovered between apoptosis and mitochondrial dynamics. During apoptosis, the mitochondria undergo a significant morphological change from a filamentous network to punctated fragments [35–37]. Mitochondrial fission participates in apoptosis through various significant mechanisms [38–40]. Tailor *et al*. found that mitochondrial fission by regulating the level of Drp-1 induce cell cycle arrest and apoptosis in human CRC cells [41]. Cardiac apoptosis-related lncRNA (CARL) can suppress mitochondrial fission and apoptosis by targeting miR-539 and PHB2 [42]. However, minimal evidence showed the link between mitochondrial fission and AECs-II apoptosis in lung fibrosis. Our data revealed that abnormal mitochondrial fission participate in AECs-II apoptosis in the progression of lung fibrosis. Li *et al*. have demonstrated that miR-30 could regulate mitochondrial fission through targeting p53 and the dynamin-related protein-1 pathway [10]. Quintavalle *et al*. have reported that miR-30b/c could regulate TRAIL-induced apoptosis in glioma cells [43]. In our present study, gain- and loss-of-function studies have demonstrated that the up/down-regulation of miR-30a could promote/inhibit mitochondrial fission and consequent apoptosis in AECs-II. And the expression of miR-30a in lung fibrosis was inversely relevant to mitochondrial fission and apoptosis *in vivo*.

Mitochondrial fission is dependent on Drp-1. Drp-1 interacts with Bax to form complexes at mitochondrial fission sites; these complexes mediate in outer mitochondrial membrane permeabilization and cytochrome c release [9]. Drp-1 is actively targeted to the outer mitochondrial membrane by non-GTPase receptor proteins, including mitochondrial fission protein 1, mitochondrial fission factor and mitochondrial elongation factor 1 [44,45]. During apoptosis, Drp-1 is recruited from the cytoplasm to the mitochondria and assembled into spiral multimeric complexes at the scission sites, where it constricts the organelles [46,47]. Many studies demonstrate that overexpression Drp-1 can induce apoptosis [48]. In the present study, Drp-1 expression increased and Drp-1 translocated from the cytoplasm to the mitochondria in lung fibrosis which is critical for AECs-II apoptosis. Several studies have demonstrated that p53 can influence Drp-1 expression [49]. MiR-30a regulates the expression of p53 [50]. Consistent with these reports, our data showed that the level of p53 was negatively correlated with miR-30a, while positively correlated with Drp-1 expression.

Several studies have suggested that a novel approach using miRNA therapeutics can treat lung fibrosis [51]. Our studies showed that miR-30a could decrease AECs-II apoptosis by repressing the mitochondrial fission dependent on Drp-1 and participate in the regulation of lung fibrosis. The data indicated that miR-30a might be a potential target for developing novel therapeutics in treating lung fibrosis.
